# Gene expression signatures of breast cancer stem and progenitor cells do not exhibit features of Warburg metabolism

**DOI:** 10.1186/s13287-015-0153-7

**Published:** 2015-08-28

**Authors:** Nicole Gordon, Amy M. Skinner, Rodney F. Pommier, Robynn V. Schillace, Steven O’Neill, Jennifer L. Peckham, Patrick Muller, Mary E. Condron, Cory Donovan, Arpana Naik, Juliana Hansen, SuEllen J. Pommier

**Affiliations:** Department of Surgery, Division of Surgical Oncology, Oregon Health & Science University, 3181 SW Sam Jackson Park Road, Mail Code L619, Portland, OR 97239 USA; Department of Surgery, Division of Plastic & Reconstructive Surgery, Oregon Health & Science University, Portland, OR USA

## Abstract

**Introduction:**

Cancers are believed to adapt to continual changes in glucose and oxygen availability by relying almost exclusively on glycolytic metabolism for energy (i.e. the Warburg effect). The process by which breast cancers sustain growth in avascular tissue is thought to be mediated via aberrant hypoxia response with ensuing shifts in glycolytic metabolism. Given their role in initiating and perpetuating tumors, we sought to determine whether breast cancer stem and progenitor cells play an instrumental role in this adaptive metabolic response.

**Methods:**

Breast cancer stem/progenitor cells were isolated from invasive ductal carcinomas, and benign stem cells (SC) were isolated from reduction mammoplasty tissues. Relative expression of 33 genes involved in hypoxia and glucose metabolism was evaluated in flow cytometrically isolated stem and progenitor cell populations. Significance between cohorts and cell populations was determined using Student’s 2-tailed t test.

**Results:**

While benign stem/progenitor cells exhibited few significant inter-group differences in expression of genes involved in hypoxia regulation or glucose metabolism, breast cancer stem/progenitor cells demonstrated significant inter-group variability. Breast cancer stem/progenitor cells adapted to microenvironments through changes in stem cell numbers and transcription of glycolytic genes. One of four breast cancer stem/progenitor cells subpopulations exhibited an aerobic glycolysis gene expression signature. This subpopulation comprises the majority of the tumor and therefore best reflects invasive ductal carcinoma tumor biology. Although PI3K/AKT mutations are associated with increased proliferation of breast cancer cells, mutations in breast cancer stem/progenitor cells subpopulations did not correlate with changes in metabolic gene expression.

**Conclusions:**

The adaptive capacity of breast cancer stem/progenitor cells may enable tumors to survive variable conditions encountered during progressive stages of cancer growth.

## Introduction

Breast cancer is the second most common malignancy diagnosed in the world and the second leading cause of cancer death among women [1]. To date, there is no cure for recurrent or metastatic disease, which remains the primary cause of death for women diagnosed with breast cancer. Elucidating mechanisms that underlie the pathogenesis of breast cancer is imperative for the purpose of developing therapies with greater efficacy.

Early biochemical studies revealed that cancer cells rapidly increase cellular proliferation by utilizing aberrant energy pathways. Instead of completing the standard steps in glucose metabolism of glycolysis, Krebs cycle, and oxidative phosphorylation, cancer cells rely heavily on glycolysis, and produce a considerable amount of lactate in the presence of adequate oxygen, a phenomenon known as the Warburg effect [2]. However, while some reports assert that cancer cells upregulate glycolysis as an adaptive response to changing oxygen requirements as the tumor grows, others contend that aerobic glycolysis is merely a reflection of rapidly dividing cells [3–5]. Some believe that rare populations of stem-like cells reside in benign adult tissues and routinely perform Warburg metabolism, and that these cells maintain this metabolic feature throughout carcinogenic transformation [6–8]. Other reports believe that a symbiotic or “reverse Warburg” relationship exists between glycolytic tumor stromal cells, which generate glycolytic byproducts, and nonglycolytic neighboring cells, which utilize these byproducts for energy [9, 10]. Still other reports claim that cancer cells shift to glycolytic metabolism as a result of specific oncogenic or tumor suppressor mutations [11, 12]. Differentiating between these models has important implications for the diagnosis, management, and treatment of cancer patients.

Recent studies have implicated cancer stem cells (CSCs) in the heterogeneity, viability, propagation, and recurrence of tumors [13]. First identified in hematologic cancer, CSCs have since been identified in many solid tumors, including breast cancer. Although breast tumors are comprised of less than 5 % breast cancer stem/progenitor cells (BCSCs), specific cellular subpopulations can be prospectively isolated from human tumors and efficiently give rise to phenotypically similar tumors in immunodeficient mice [14]. Despite the fact that CSCs are recognized as an essential component of tumors, the adaptive mechanisms of malignant transformation employed by BCSCs are not thoroughly understood.

We hypothesized that BCSCs modulate tumor fitness via influencing the behavior of the more differentiated cell components of the tumor. The BCSCs may be metabolically reprogramming nonstem breast tumor cells toward a glycolytic state. To test this hypothesis, BCSCs from invasive ductal carcinoma (IDC) tissues and benign breast stem/progenitor cells (SCs) from reduction mammoplasty tissues were flow cytometrically isolated and analyzed for differences in expression of 33 genes involved in hypoxia response and glucose metabolism.

## Materials and methods

### Cell culture

Human HCC1937, HCC1806, MCF7, and MCF10A cells were acquired from ATCC (Manassas, VA, USA). Cells were cultured at subconfluence, utilized at low passage level, and maintained according to ATCC recommendations.

### Patient selection and surgical specimens

After obtaining Oregon Health & Science Institutional Review Board (OHSU IRB) approval and patient consent, tissues for this study were collected from 15 patients with IDC and 11 patients who underwent reduction mammoplasty. All procedures were followed in accordance with the ethical standards of the OHSU IRB and the Declaration of Helsinki. All tissues were subject to pathologic evaluation and confirmation of diagnosis.

### Isolation of cell populations

Fresh breast tissue was obtained from pathology, finely minced, and immediately cryopreserved in liquid nitrogen. Upon preparation for analysis, tissue specimens were thawed, and digested overnight in a Pyrex (nonadherent) trypsinizing flask with mammary epithelial cell-specific medium containing collagenase/hyaluronidase (Epicult; StemCell Technologies, Vancouver, BC, Canada). Cells were agitated on a rotary shaker overnight, with incubation at 37 °C. The following day, all cells were washed with phosphate-buffered saline + 2 % fetal bovine serum, pelleted, and digested with DNase and trypsin. SCs and BCSCs were immediately isolated (without culture) via fluorescence-activated cell sorting (FACS) using a BD Influx or BD Vantage sorter (BD Biosciences, San Jose, CA, USA) and CellQuest software (San Jose, CA, USA). Antibodies were obtained from BD Biosciences unless otherwise stated. Lineage committed endothelial (CD31-FITC^+^), lymphocyte (CD45-FITC^+^), and nonviable (PI^+^) populations were removed, and CD49f-APC (BD Bioscience, San Jose, CA, USA) and CD24-PE were used to sort cells into the following populations: CD49f^hi^CD24^hi^, CD49f^low^CD24^hi^, CD49f^hi^CD24^low^, and CD49f^low^CD24^low^. Additional population statistics were collected based on gating with CD44-PECy7^+^ and EpCAM-brilliant violet^+^ (BioLegend, San Diego, CA, USA).

### Hypoxia/normoxia cell culture

Equal numbers of subconfluent cells (T47D, MCF7, HCC1937, HCC1806, and MCF10A) were plated in six-well tissue culture plates in corresponding media. Additionally, a subset of cells digested from benign tissue and cancer specimens were cultured at 37 °C overnight in hypoxic (1 % oxygen) or normoxic (21 % oxygen) conditions prior to FACS. Hypoxia was administered by flushing a gas-impermeable chamber (Billups-Rothenberg, Inc., Del Mar, CA, USA) with 1 % oxygen. All cells (suspension and adherent) were harvested, and were then stained and sorted into corresponding BCSC or SC subpopulations.

### TaqMan low-density array

RNA was extracted from whole tissue or sorted cell populations using a Qiagen RNeasy kit (Germantown, MD, USA), and reverse transcribed using a Superscript III First-Strand Synthesis System with random hexamer primers (Life Technologies, Waltham, MA, USA). Expression of 33 hypoxia and glucose metabolism-related genes (HypoxyGluMet) was measured by quantitative RT-PCR via a low-density array (TLDA; Life Technologies) microfluidic card according to the manufacturer’s protocol. Differences in gene expression were determined using Expression Suite v1.0.2 software (Life Technology, Waltham, MA, USA). Genes with undetermined cycle threshold (Ct) scores were reassigned a delta Ct value of 40 if the amplification score for the endogenous gene and the gene of interest (18S) was ≥0.80. Gene Card 1 assayed the following genes: *ALDOA* [UniGene:Hs.513490], *AKT1* [UniGene:Hs.525622], *ENO1* [UniGene:Hs.517145], *EPO* [UniGene:Hs.2303], *GSK3B* [UniGene:Hs.445733], *HIF1A* [597216], *HMOX1* [UniGene:Hs.517581], *LDHA* [UniGene:Hs.5795], *LOX* [UniGene:Hs.102267], *PDHA1* [UniGene:Hs.530331], *PFKL* [UniGene:Hs.255093], *PGK1* [UniGene:Hs.78771], *PKLR* [UniGene:Hs.95990], *PKM2* [UniGene:Hs.534770], *PLOD2* [UniGene:Hs.477866], *SLC2A1* [UniGene:Hs.473721], *TFRC* [UniGene:Hs.529618], and *VEGFA* [UniGene:Hs.73793]. Gene Card 2 assayed the following genes: *EGR1* [UniGene:Hs.326035], *G6PD* [UniGene:Hs.461047], *GAPDH* [UniGene:Hs.544577], *HK2* [UniGene:Hs.406266], *SLC16A3* [UniGene:Hs.500761], *NGFR* [UniGene:Hs.415768], *PC* [UniGene:Hs.89890], *PCK2* [UniGene:Hs.75812], *PDK1* [UniGene:Hs.470633], *PPARGC1A* [UniGene:Hs.527078], *SLC2A3* [UniGene:Hs.419240], *SLC9A1* [UniGene:Hs.469116], *SOD2* [UniGene:Hs.487046], *TALDO1* [UniGene:Hs.438678], and *TKT* [UniGene:Hs.89643].

### Statistical analysis

Unless otherwise stated, statistical significance was determined by Student’s unpaired *t* test with two-tailed distribution.

## Results

### Breast cancer cell lines convert to glycolytic metabolism following hypoxia

**Fig. 1:**
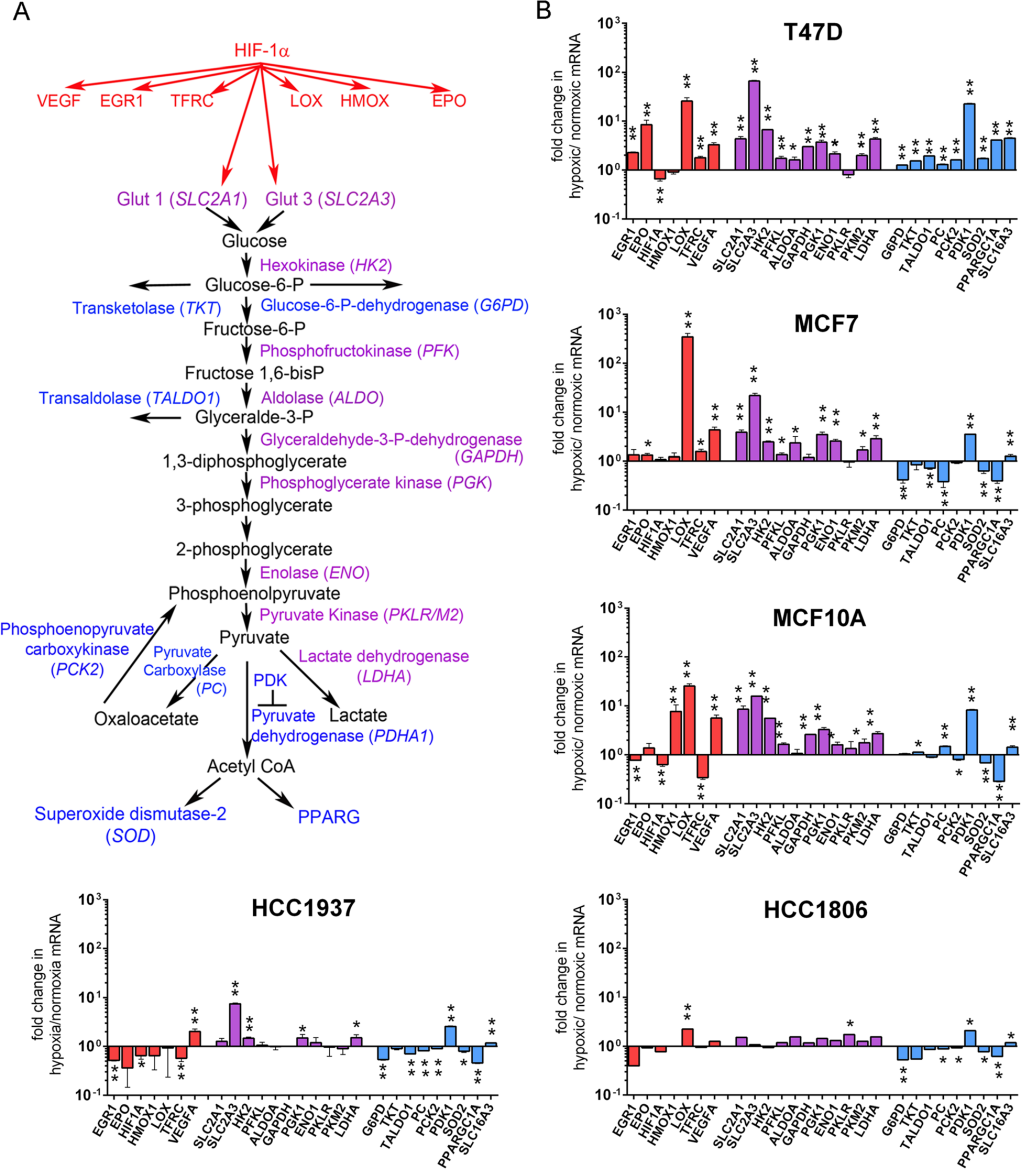
Validation of the HypoxyGluMet gene expression array in breast cancer cell lines. **a** Overview of HIF-1-gene targets (*red*), HIF-1α-dependent glycolytic gene targets (*purple*), and HIF-1α-independent glycolytic gene targets (*blue*) assayed by the HypoxyGluMet array. For clarity and consistency, this color scheme is employed in each figure. Gene symbols are shown in parentheses. **b** Breast cancer cell lines T47D, MCF7, HCC1806, HCC1937, and benign epithelial MCF10A cells were cultured overnight under hypoxic or normoxic conditions. RNA was isolated, cDNA was synthesized, and relative expression of HIF-1 nonglycolytic gene targets (*red columns*), HIF-1**-**dependent glycolytic gene targets (*purple columns*), and HIF-1**-**independent glycolytic gene targets (*blue columns*) was quantified via the HypoxyGluMet array. Data shown are the mean of two independent experiments; individual samples were performed in duplicate (Gene Card 2, see Materials and methods) or in triplicate (Gene Card 1). Significance was determined using Student’s paired *t* test on ΔCt values, with two-tailed distribution. Error bars were calculated as the standard error of the mean (SEM). *p ≤0.05, **p ≤0.01

To better understand the respective roles of hypoxia and glycolysis in driving the malignant transformation of BCSCs, a novel gene expression array (HypoxyGluMet) was designed. Hypoxia inducible factor-1α (*HIF-1α*) is a transcription factor orchestrating cellular survival in response to hypoxic conditions, including induction of several glycolysis genes. Glycolytic and nonglycolytic *HIF-1α* gene targets were examined in the array (Fig. 1a). To validate the assay, the metabolic profile of well-characterized breast cancer cell lines and MCF10A mammary epithelial cells was examined after 24 hours of culture under normoxic (21 %) or hypoxic (1 %) conditions. Consistent with published observations, gene expression of *HIF-1α* remained relatively unaffected (i.e., <2-fold difference) by hypoxic culture conditions in each of the cell lines examined, while *HIF-1α* inducible genes were affected by hypoxia (Fig. 1b) [15]. Notably, in response to hypoxic conditions, the relative expression of lysyl oxidase (*LOX*) significantly increased 25-fold and 350-fold in T47D and MCF7 cell lines, respectively (*p* <0.0001, Fig. 1b). The relative expression of vascular endothelial growth factor A (*VEGFA*) in hypoxia-cultured cells was also significantly increased in four of five of the cell lines (*p* ≤0.005), compared with normoxic-cultured controls. Most of the cell lines demonstrated a significant change in relative expression of each gene target induced by *HIF-1α* in response to hypoxic conditions. Although the array examined expression of 33 genes, only genes exhibiting statistical significance in at least one cell line are shown in each figure. See Materials and methods for a complete list of the genes examined.

To further validate the HypoxyGluMet array, normoxic or hypoxic-cultured breast cancer cell lines and MCF10A mammary epithelial cells were evaluated for changes in expression of HIF-1α-induced glycolytic genes. Expression of the glucose transporter 3 gene (*SLC2A3*), which is translated into glucose transporter protein GLUT3, was most significantly augmented in response to hypoxic conditions, with a 22-fold to 62-fold relative increase observed in MCF7 and T47D cells, respectively (*p* ≤0.0016, Fig. 1b). Observation of significantly elevated expression of the majority of HIF-1α-dependent glycolytic gene targets with concurrent divergent patterns in expression of HIF-1α-independent glycolytic gene targets in each of the hypoxia treated cell lines supports the validity of employing the HypoxGlutMet array to elucidate the metabolic profile of BCSCs.

### HIF-1α gene targets are overexpressed in fresh IDC specimens

**Fig. 2:**
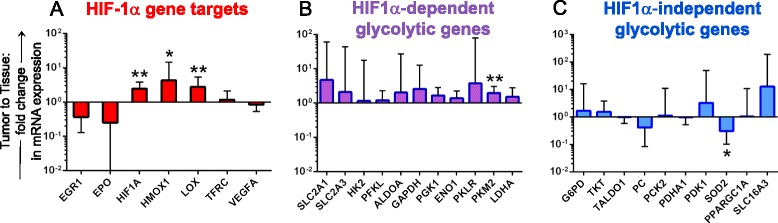
Relative expression of glycolytic genes in breast tumor versus benign tissue. The HypoxyGluMet array was employed on tumors (*n* = 15) and benign tissues (*n* = 11) to determine relative expression of **a** HIF-1 and HIF-1-dependent nonglycolytic genes, **b** HIF-1α-dependent glycolytic genes, and **c** HIF-1α-independent glycolytic genes. Average gene expression in tumor tissues is shown relative to average expression in benign tissues. Error bars represent the 95 % confidence interval. *p ≤0.05, **p ≤0.01

Based on reports of increased glyceraldehyde-3-phosphate dehydrogenase, pyruvate kinase, and lactate dehydrogenase in breast carcinomas, we hypothesized that glycolytic metabolism genes would be increased in primary breast cancer specimens [16–18]. Indeed, when gene expression analysis was conducted on fresh surgical specimens, the HypoxyGluMet array revealed that breast tumors exhibited a 2.5-fold increase in expression of HIF-1α compared with benign tissues (Fig. 2a). Significant increases were also observed in expression of HIF-1α targets, heme oxygenase 1 (*HMOX1*; fourfold increase, *p* = 0.003) and *LOX* (2.8-fold increase, *p* = 0.005). Several *HIF-1α*-inducible glycolytic genes were overexpressed in breast tumors, although most of these increases did not reach statistical significance (Fig. 2b). Pyruvate kinase isozyme M2 (*PKM2*) was the exception, in that it was expressed significantly higher (twofold, *p* = 0.004) in breast tumors compared with benign tissues.

Glycolytic genes not known to be dependent upon HIF-1α induction demonstrated similar expression between breast cancer specimens and benign tissues (Fig. 2c). Expression of the mitochondrial gene superoxide dismutase 2 (*SOD2*) was included in the HypoxyGluMet array because SOD2 is expressed in mitochondria where reactive oxygen species (ROS) are generated in response to hypoxia or respiratory bursts of the electron transport chain [19–21]. Consistent with published observations of decreased SOD2 expression in many cancers, we observed threefold lower expression of SOD2 in cancer specimens compared with benign breast tissue (*p* = 0.03, Fig. 2c).

### BCSCs and SCs exhibit distinctive metabolic profiles

We have previously isolated and characterized stem and progenitor cells from human breast tissue by employing cell surface markers CD49f (integrin α6) and CD24 [22–26]. Abundant heterogeneity exists between cell populations, breast cancers, and patients, so the definitive immunophenotype of breast CSCs has not yet been reconciled; however, other studies in the CSC field have similarly employed these markers to isolate and characterize human breast stem and progenitor cells [27–33].

**Fig. 3:**
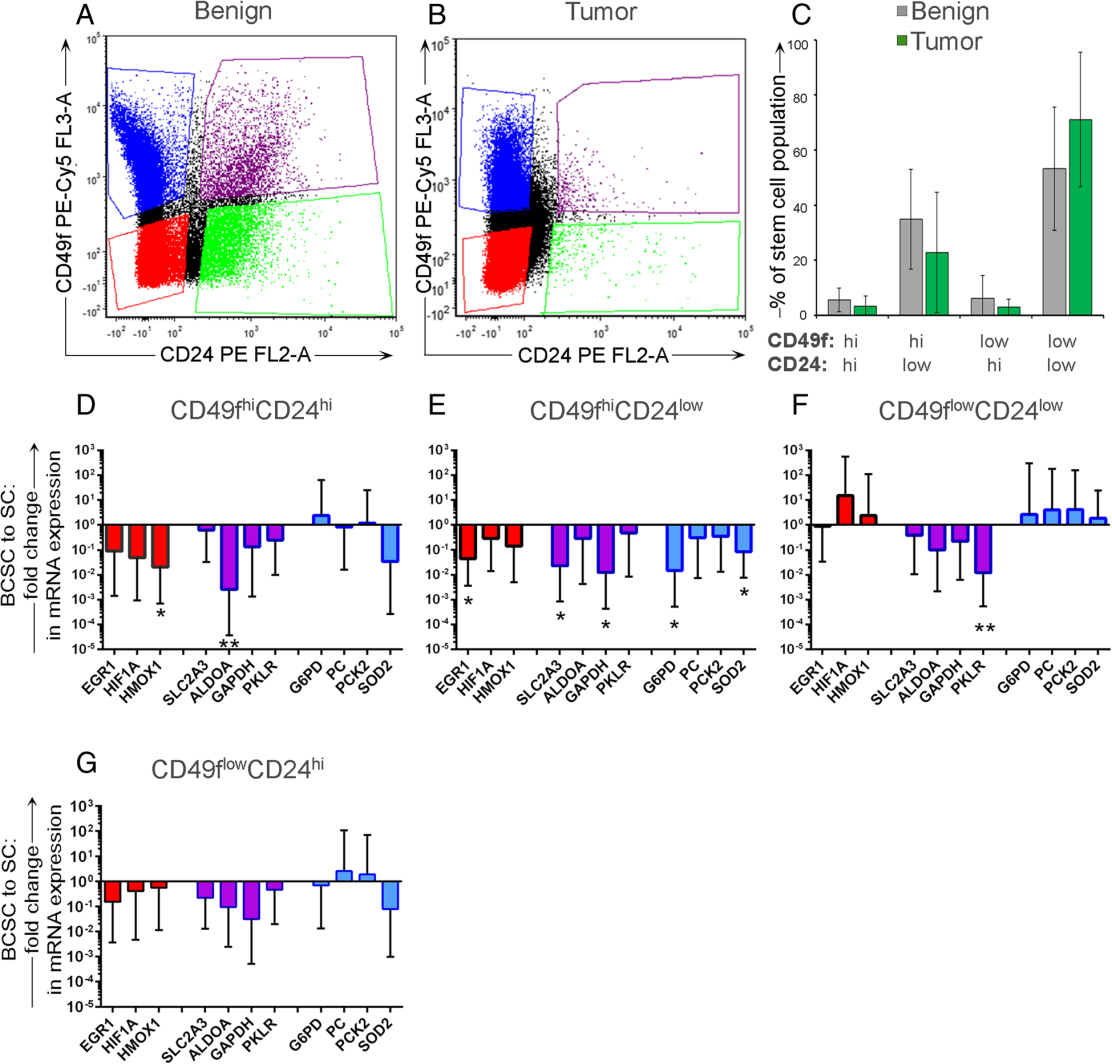
BCSCs exhibit decreased expression of several metabolic genes relative to SCs. **a**, **b** Representative FACS plots of subpopulations isolated from **a** benign or **b** malignant breast tissue. **c** Average percentage of total CD45^−^, CD31^−^ (lin^–^) stem, and progenitor subpopulations isolated from benign (*gray*, *n* = 15) or malignant (*green*, *n* = 11) breast tissue. Error bars represent standard deviation of the mean. **d**–**g** Gene expression was determined for subpopulations of BCSCs (*n* = 13) relative to SC counterparts (*n* = 9). HIF-1α and nonglycolytic HIF-1α-inducible gene targets (*red*), HIF-1α-dependent glycolytic gene targets (*purple*), and HIF-1α-independent glycolytic gene targets (*blue*) are color coded. Error bars represent the 95 % confidence interval

Having examined stem and progenitor cell quality and quantity in CD49f and CD24 variably expressing populations using a TaqMan Human Stem Cell Pluripotency Array Micro Fluidic Card and surrogate mammosphere assays in previous work [23], we herein sought to directly compare metabolic profiles of these stem and progenitor-like cell subpopulations between breast cancer specimens and benign breast tissues. BCSCs from 15 IDC tissues and SCs from 11 reduction mammoplasty tissues were isolated via FACS to exclude lineage-committed populations (CD31 and CD45) and collect CD49f and CD24 cells. Subpopulations expressing CD49f^hi^CD24^low^, CD49f^hi^CD24^hi^, CD49f^low^CD24^hi^, and CD49f^low^CD24^low^ were isolated (Fig. 3a, b). Consistent with our previous observations, we observed substantial quantitative differences in the number of cells in each population; although we observed variability between samples, the percentages of BCSCs isolated from tumors did not statistically differ from the percentages of SCs isolated from benign tissues (Fig. 3c) [23].

The HypoxyGluMet array was employed to characterize each cellular subpopulation. Contrary to the expectation that *HIF-1α* would be activated by glycolytic metabolites in cancer cells [34], we observed that CD49f^hi^ BCSCs (CD49f^hi^CD24^hi^, CD49f^hi^CD24^low^) demonstrated reduced expression of HIF-1α gene targets as well as of HIF-1α-inducible glycolytic genes compared with CD49f^hi^ SCs (Fig. 3d, e). Specifically, significant decreases in expression of *HMOX1* (−50-fold, *p* = 0.03) and aldolase (*ALDOA*; −500-fold, *p* = 0.01) were observed in CD49f^hi^CD24^hi^ cancer cells compared with benign cell counterparts. Similarly, CD49f^hi^CD24^low^ tumor cells exhibited significantly lower expression of *EGR1* (−20-fold, *p* = 0.02), glucose transporter 3 (*SLC2A3*; −50-fold, *p* = 0.03), and *GAPDH* (−100-fold, *p* = 0.01) than benign cell counterparts.

There were few differences in HypoxyGluMet gene expression profiles between subpopulations of CD49f^low^ cells (CD49f^low^CD24^hi^, CD49f^low^CD24^low^) isolated from cancer versus benign breast tissue. However, CD49f^low^CD24^low^ BCSCs exhibited significantly reduced expression of PKLR (−100-fold, *p* = 0.01) compared with SC counterparts (Fig. 3f). There were no significant differences detected in gene expression between CD49f^low^CD24^hi^ BCSCs versus SC counterparts (Fig. 3g).

### Glycolytic metabolism gene transcripts are lower in BCSCs than in tumor of origin

**Fig. 4:**
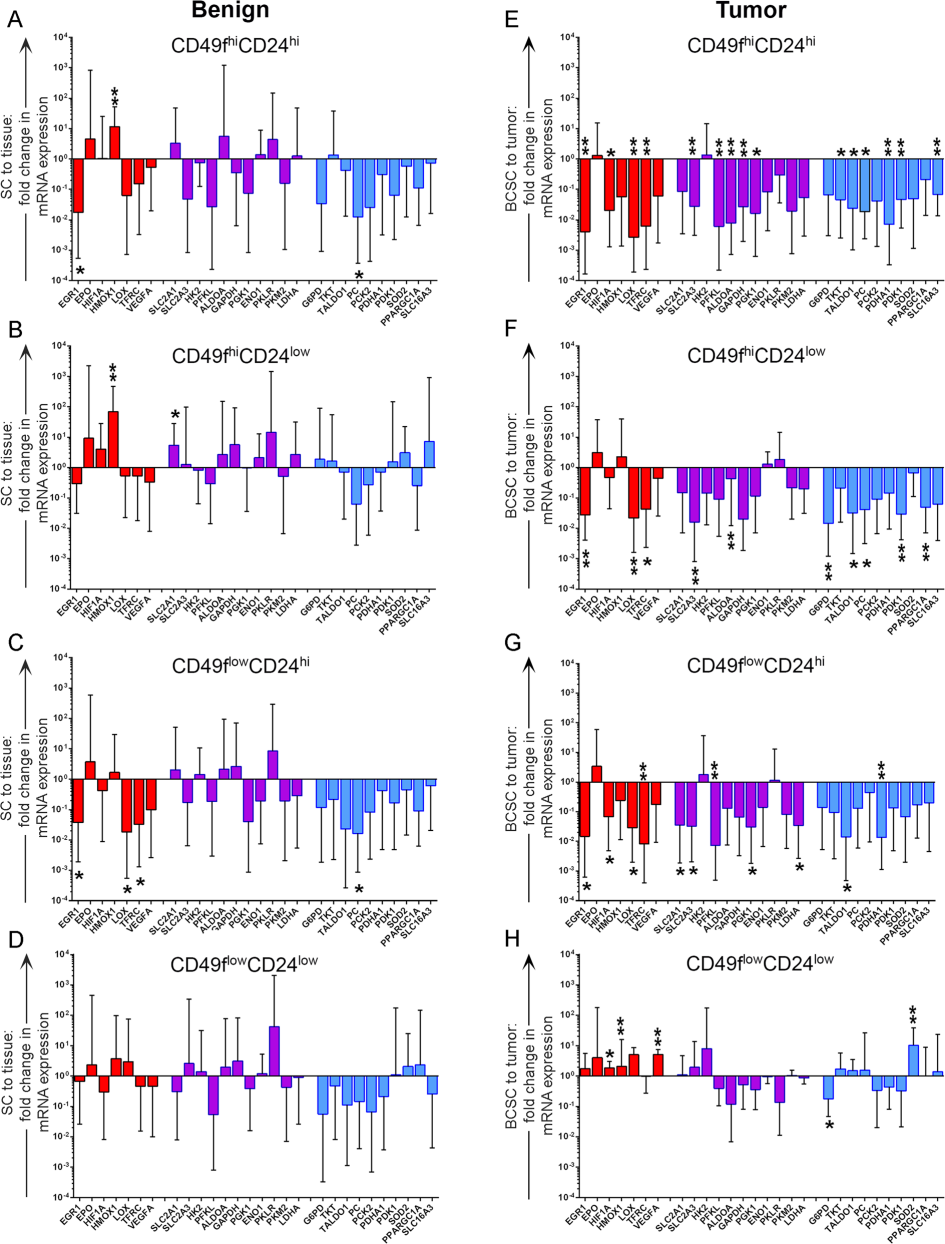
Metabolic signature of BCSC and SC subpopulations. **a**–**d** Gene expression in subpopulations of SCs was determined relative to corresponding benign tissue (*n* = 10). **e**–**h** Gene expression in subpopulations of malignant BCSCs was determined relative to corresponding tumor (*n* = 11). HIF-1α and nonglycolytic HIF-1α-inducible gene targets (*red*), HIF-1α-dependent glycolytic gene targets (*purple*), and HIF-1α-independent glycolytic gene targets (*blue*) are color coded. Significance was determined by Student’s paired *t* test with two-tailed distribution. Error bars represent the 95 % confidence interval. *p ≤0.05, **p ≤0.01 *BCSC* breast cancer stem/progenitor cell, *SC* benign breast stem/progenitor cell

When compared with corresponding tissue from which stem cells were obtained, SCs exhibited few significant intergroup differences in HypoxyGluMet gene expression (Fig. 4a–d). One exception, however, is expression of *HMOX1* in CD49f^hi^ benign subpopulations, which was augmented 12-fold (*p* = 0.005) and 69-fold (*p* = 0.0006) in CD49f^hi^CD24^hi^ and CD49f^hi^CD24^low^ SC populations relative to tissue, respectively.

In contrast, BCSCs demonstrated significant intergroup variability. Three of four BCSC subpopulations exhibited lower overall expression of glucose metabolism genes than the tumor of origin (Fig. 4e–h). The CD49f^hi^CD24^hi^ subpopulation most significantly differed from the tumor of origin, with reduced expression observed in the majority of HIF-1α inducible glycolytic genes, as well as HIF-1α-independent glycolytic genes (Fig. 4e). Although the CD49f^low^CD24^low^ BCSC subpopulation exhibited nearly twofold increased expression of HIF-1α (*p* = 0.02) compared with the tumor of origin, with corresponding increases in expression of downstream HIF-1α-dependent genes (*LOX, VEGFA*), we did not observe significant differences in the expression of HIF-1α-dependent glucose metabolism genes (Fig 4h). This subpopulation exhibited gene expression most similar to that of unfractionated tumor of origin.

### BCSCs do not increase glycolytic gene expression in response to hypoxia

**Fig. 5:**
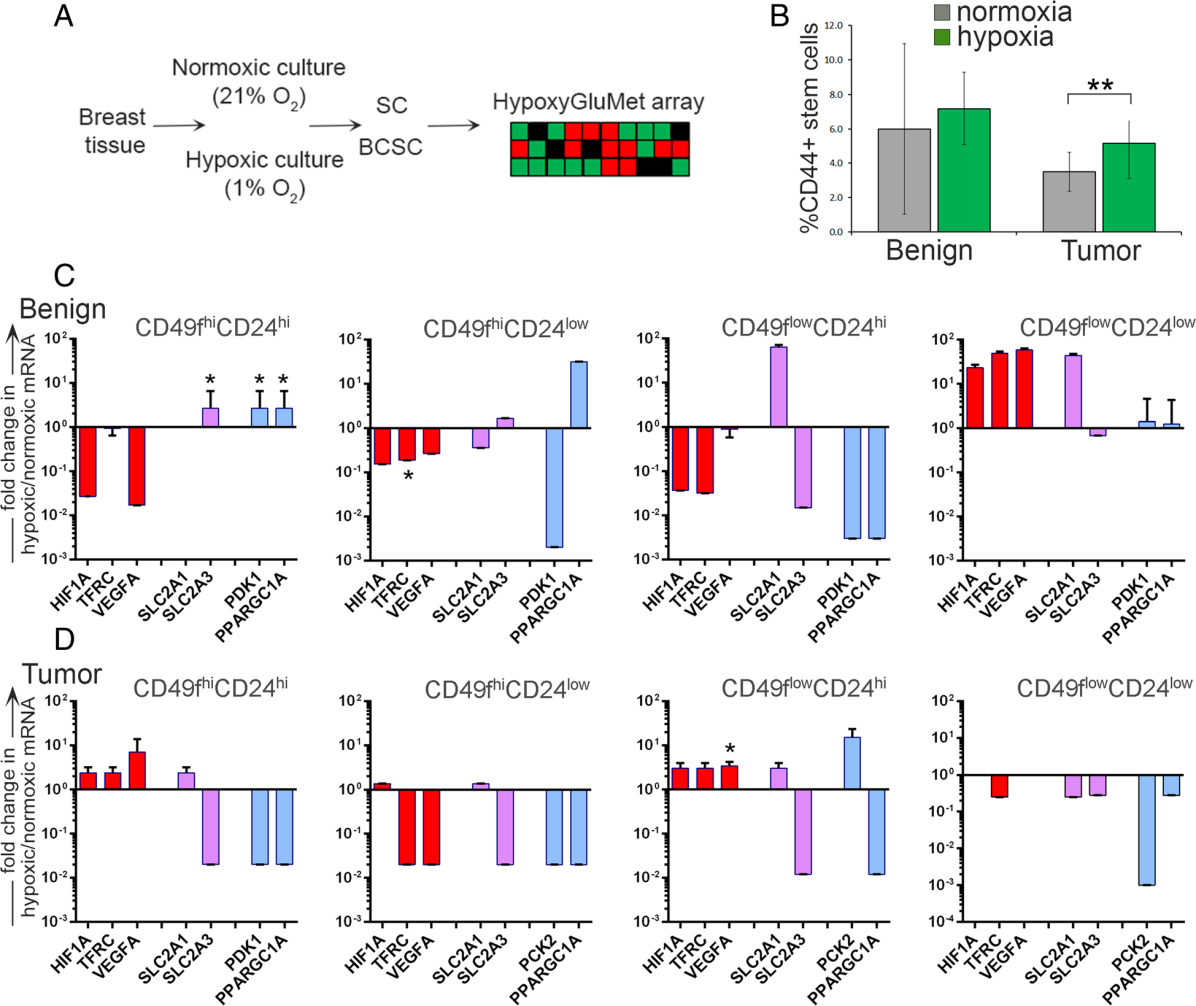
Differential response of BCSCs and SCs to hypoxia. **a** Schematic of experimental design. **b** Breast cancer (*n* = 2) and benign tissue (*n* = 2) specimens were enzymatically digested into single cell suspension. Cells were divided equally and cultured overnight in normoxic or hypoxic conditions. BCSC and SC populations (CD49f^+^CD24^+^, CD49f^+^CD24^−^, CD49f^−^CD24^+^, CD49f^−^CD24^−^) were immediately isolated by FACS analysis of all cells (adherent and suspension) the following day, and gene expression was determined via the HypoxyGluMet array. Error bars represent of two samples, run in duplicate (Gene Card 1) or in triplicate (Gene Card 2). Significance was determined by Student’s paired *t* test with two-tailed distribution. **p <0.01. **c**, **d** Gene expression of HIF-1α-dependent genes (*red*), HIF-1α-dependent glycolytic genes (*purple*), and HIF-1α-independent glycolytic genes (*blue*) was determined in hypoxic-treated relative to normoxic-treated **c** benign and **d** tumor specimens. Error bars (present in all panels but possibly too small to visualize) represent standard deviation of the mean. Significance was determined by Student’s paired *t* test with two-tailed distribution. *p ≤0.05. *BCSC* breast cancer stem/progenitor cell, *SC* benign breast stem/progenitor cell

To determine how acute changes in oxygen availability affect HIF-1α-dependent gene expression and cellular metabolism in primary BCSCs, single cell suspensions were prepared from breast cancer specimens and cells were cultured overnight under hypoxic or normoxic conditions (Fig. 5a). The following day, all cells (in suspension or attached) were sorted into BCSC subpopulations. The hyaluronan receptor, CD44, is implicated in cancer cell growth and proliferation, and is induced by hypoxia in triple-negative breast cancer [35–37]. Expression of CD44 was therefore assessed in each BCSC subpopulation. There was a 1.5-fold increase in the proportion of cells coexpressing CD44^+^ and BCSC markers following overnight culture in hypoxic versus normoxic conditions (Fig. 5b). Similar amplification of SCs expressing CD44 following hypoxia was observed but was not significant.

To further investigate how BCSCs respond to hypoxia, subpopulations were isolated from hypoxia-exposed and normoxia-exposed cells via FACS, RNA was isolated from each population, and gene expression was quantified using the HypoxGluMet array. Benign specimens were treated and isolated with similar approach. In SCs, there were few significant differences in metabolic gene expression following hypoxia culture (Fig. 5c). Genes encoding glucose transporter 3 (*SLC2A3*), pyruvate dehydrogenase kinase (*PDK*), and peroxisome proliferator-activated receptor gamma coactivator 1-α (*PPARGC1A*) were augmented nearly threefold in CD49f^hi^CD24^hi^ cells (Fig. 5c). In hypoxia-cultured BCSCs, the CD49f^low^CD24^hi^ subpopulation demonstrated a threefold increase in the gene encoding vascular endothelial growth factor (*VEGF*), and was the only significant difference observed (Fig. 5d). There were no significant differences observed in CD49f^low^CD24^low^ subpopulations isolated from benign or malignant specimens.

### No correlation with *PIK3CA* mutation or hormone receptor status

When wild-type cells encounter hypoxic conditions, phosphatidylinositol 3-kinase (PI3K) phosphorylates Akt, resulting in inactivation of glycogen synthase kinase GSK-3 and subsequent decrease in cellular metabolism and proliferation. If the PI3K/AKT pathway is impaired, as frequently occurs in cancer, cells are subject to increased glycolysis and proliferation despite low oxygen availability [38–40]. We previously described the mutational profile of 30 IDC specimens (including all 15 tumors from this study) and corresponding BCSC subpopulations using Sequenome Mass ARRAY analysis [22]. Of the 15 tumors subject to glycolytic metabolic profiling, nine patients were identified with tumor and/or BCSC mutations in the catalytic subunit encoding the PI3K enzyme *PIK3CA*. Examination of whether the PI3K/AKT mutations correlated with changes in tumor or BCSC metabolic gene expression observed in the HypoxyGluMet array did not identify any predictive differences (data not shown).

In a previous study that utilized immunohistochemistry to characterize the metabolic phenotype of 740 breast cancers, a greater association of Warburg metabolism was observed in breast cancers that lack expression of hormone receptors (estrogen receptor (ER^−^), progesterone receptor (PR^−^), and human epidermal growth factor 2 (HER2^−^)) [41]. Similarly, another group performed a proteomic analysis on breast tumors and observed augmented glycolytic protein content in ERα^−^ tumors relative to ERα^+^ tumors [42]. We similarly assessed whether changes in BCSC gene expression we observed correlated with ER status but did not observe any significant associations (data not shown). There were also no notable correlations between gene expression patterns and size of tumor, receptor status (e.g., ER, PR, or Her2/neu), or tumor stage (data not shown).

## Discussion

In benign tissues, the principle means of cellular adaptation to low oxygen availability is via HIF-1α-mediated induction of several genes that act in concert to decrease cell proliferation, decrease the rate of glycolysis, and increase angiogenesis in response to increased demand for oxygen [40]. Cancer cells often usurp the HIF-1 pathway to facilitate cell growth, survival, and angiogenesis as rapidly growing cells continually deplete available oxygen from tissue. Byproducts of glucose metabolism, such as pyruvate and lactate, stabilize HIF-1α, and further induce other HIF-1α-responsive genes [34]. Given their significant role in propagating and perpetuating tumors, we sought to determine whether BCSCs adapt to a hypoxic microenvironment by adopting a glycolytic (i.e., Warburg-like) state.

We designed a HypoxyGluMet gene expression array to survey the expression of genes activated by HIF-1α as well as genes involved in glucose metabolism. We employed the array to compare gene expression profiles between breast cancers and benign breast tissues and determined that there were few significant differences between tissues. Increased expression of *PKM2* was observed in tumors as expected because the *PKM2* isoform is only expressed in cancer cells, stem cells, or embryonic stem cells [43]. On the contrary, when gene expression profiles of BCSC subpopulations were directly compared with profiles of SC counterparts, we observed significantly greater differences (i.e., up to 1000-fold) in gene expression in three of four subpopulations. The population most divergent from the benign counterpart was the CD49f^hi^CD24^low^ subpopulation, which demonstrated reduced expression in the majority of assayed transcripts. The CD49f^low^CD24^hi^ subpopulation did not exhibit any statistically significant differences in profiles. It is possible that stem cell hierarchy played a role in the profile differences observed between SC and BCSC populations, because considerable differences in glycolytic metabolism have been observed and attributed to CSC hierarchy in several breast cancer cell lines [44].

Somatic stem cells, such as long-term repopulating hematopoietic stem cells (LT-HSC), neural stem cells, and mesenchymal stem cells maintain quiescence by inhabiting a hypoxic niche to impart stringent control of HIF-1α, and utilizing ROS signaling to progress to a proliferative or differentiated state [45]. As HIF-1α is active in quiescent stem cells and induces expression of several glycolytic genes, somatic stem cells accordingly exhibit a proglycolytic phenotype. Our data reveal that SCs similarly exhibit proglycolytic traits, as expression of several HIF-1α-dependent genes (glycocytic and nonglycocytic) was increased relative to corresponding tissue. SCs demonstrating features of glycolytic metabolism is further supported by increased expression of glucose transporter 1 and/or 3 (*SLC2A1*, *SLC2A3*) in each of the SC subpopulations relative to corresponding tissue.

Compared with the proglycolytic phenotype observed in SCs, our data revealed a considerably different metabolic profile in BCSCs. Expression of glucose transporters was decreased in three of four BCSC subpopulations relative to tumor, and expression of the glycolytic rate-limiting enzyme phosphofructokinase (*PFKL*) was decreased in all BCSC subpopulations. Moreover, transcripts of HIF-1α and corresponding gene targets (glycocytic and nonglycocytic) were reduced in three of four BCSC subpopulations relative to tumor. These results oppose the model of a classic proglycocytic phenotype. Highlighting the heterogeneous nature of tumors, the CD49f^low^CD24^low^ cells comprised a distinctive BCSC population. These cells displayed augmented expression of HIF-1α and HIF-1α-inducible nonglycocytic targets, along with minor increases in the expression of glucose transporters *SLC2A1* and *SLC2A3*. This subpopulation exhibited a gene expression profile most similar to the tumor of origin, which may be due to overrepresentation of these cells within the tumor [23]. In contrast to other BCSC subsets, the CD49f^low^CD24^low^ cells displayed features more consistent with classic Warburg metabolism.

Recent studies in tumor–microenvironment interactions describe a reverse Warburg effect in which glycolytic stromal cells nourish nonglycolytic tumor cells with byproducts of glycolytic metabolism: lactic acid and ketones [9, 10]. A comprehensive metabolic analysis of nearly 1000 breast tumors revealed that the reverse Warburg phenomenon is most commonly associated with nonaggressive luminal A tumors [41]. Similarly, we observed that a reverse Warburg phenomenon occurs between complimentary phenotypes of glycocytic and nonglycocytic BCSC subsets; however, we did not observe an association between the metabolic profile and hormone status. Discrepancies between that study and ours may be attributed to the comprehensiveness of metabolic targets assayed in our array, as well as our smaller patient cohort. Additional dissimilarities exist between cell populations examined (BCSCs versus nonspecific stromal and tumor cells).

Cells with CSC characteristics are enriched in breast cancer cell lines following repeated exposure to hypoxic conditions [46]. Although HIF-1α and corresponding inducible targets are lower in BCSCs relative to tumor at baseline, CD49f^hi^CD24^hi^ and CD49f^low^CD24^hi^ cell types expressed higher levels of these transcripts following exposure to hypoxia, as expected. In addition, expression of glucose transporter *SLC2A1* was slightly increased in three of four subpopulations. It is recognized that the magnitude of increase was not significant in the majority of targets assessed. However, when the metabolic profile of hypoxia/normoxia-treated SCs was compared with the profile of hypoxia/normoxia-treated mature populations (CD49f^hi^CD24^hi^ and CD49f^low^CD24^hi^), substantial differences covering several orders of magnitude were discerned. These data suggest that although under normal conditions BCSCs may not exhibit classic Warburg metabolism, in hypoxic conditions BCSCs can modulate tumor fitness via changes in stem cell numbers and metabolic reprogramming of nonstem breast tumor cells toward a glycolytic state, which is consistent with our initial hypothesis.

## Conclusions

Nearly a century has elapsed since Otto Warburg observed that cancer cells preferentially utilize “aerobic glycolysis” (i.e., Warburg metabolism). Recent advances in understanding the role of CSCs in the propagation and progression of tumors motivated us to characterize the metabolic profile of BCSCs. We have demonstrated that while tumors and benign tissues display similar metabolic profiles, BCSCs and SCs display distinctive profiles. Moreover, the heterogeneous nature of BCSC subpopulations supports a model of reverse Warburg metabolism in which nonglycolytic primitive (i.e., stem-like) cells may be nourished by more mature glycolytic cells in normoxic conditions. However, when rapidly expanding tumor cells outgrow their blood supply and the tissue becomes hypoxic, BCSCs can switch to a proglycocytic phenotype. The adaptive metabolic abilities of BCSCs may ensure that tumors can survive the variable conditions encountered during progressive stages of tumor growth. We propose that coupling inhibitors of hypoxia and nonglycolytic targets may offer improved therapeutic response in the treatment of breast cancer via eradication of cancer stem and progenitor cell populations.

## Abbreviations

BCSC: Breast cancer stem/progenitor cell
CSC: Cancer stem cell
Ct: cycle threshold
ER: Estrogen receptor
FACS: Fluorescence-activated cell sorting
HER2: Human epidermal growth factor 2
HIF-1α: Hypoxia inducible factor 1α
IDC: Invasive ductal carcinoma
LT-HSC: Long-term repopulating hematopoietic stem cells
OHSU IRB: Oregon Health & Science Institutional Review Board
PI3K: phosphatidylinositol 3-kinase
PR: Progesterone receptor
ROS: Reactive oxygen species
SC: Benign breast stem/progenitor cell
